# Presumed respiratory syncytial virus and severe acute respiratory syndrome coronavirus-2 co-infection in a critically ill infant: Diagnostic uncertainty and emergency management

**DOI:** 10.1017/cem.2020.445

**Published:** 2020-07-08

**Authors:** Brett Burstein, Marie-Astrid Lefebvre, Farhan Bhanji

**Affiliations:** * Division of Pediatric Emergency Medicine, Department of Pediatrics, Montreal Children's Hospital, McGill University Health Centre, Montreal, QC; † Department of Epidemiology, Biostatistics and Occupational Health, McGill University, Montreal, QC; ‡ Division of Pediatric Infectious Diseases, Infection Prevention and Control, Department of Pediatrics, Montreal Children's Hospital, McGill University Health Centre, Montreal, QC; § Division of Pediatric Critical Care Medicine, Department of Pediatrics, Montreal Children's Hospital, McGill University Health Centre, Montreal, QC

**Keywords:** Bronchiolitis, COVID-19, pediatric emergency medicine

## INTRODUCTION

On March 11, 2020, the World Health Organization declared the coronavirus disease 2019 (COVID-19) a global pandemic, with over 10,000,000 cases and 500,000 deaths worldwide by July. Under 5% of COVID-19 infections are among children, most of whom remain asymptomatic or experience mild disease.^1^ Critical illness among children is very rare, with only 48 testing positive for COVID-19 and requiring intensive care across 46 North American pediatric centres.^2^ Studies describing the epidemiological and clinical characteristics of COVID-19 have not addressed the diagnostic and management-specific challenges unique to critically ill pediatric patients. We present the case of a critically ill infant with presumed COVID-19, presenting with a clinical picture of bronchiolitis. This case highlights that, while exercising precautions for COVID-19 in critically ill children, it is essential to consider other more likely diagnoses.

## CASE PRESENTATION

**Figure 1.**
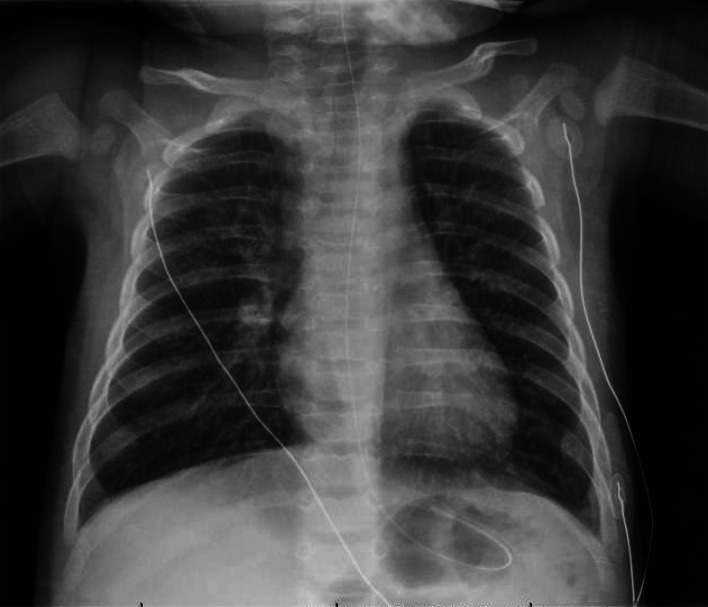
Posteroanterior chest radiograph, March 22, 2020 (Illness Day 6, Hospital Day 2); increased interstitial markings with some linear and granular opacities noted bilaterally without confluent airspace opacities.

On March 20, a 4-month-old girl was brought to a community emergency department with 5 days of cough, poor feeding, and fever. There was an afebrile 2-year-old sibling with nasal congestion but no contact with known COVID-19-infected individuals. The patient presented clinical features of bronchiolitis and was hemodynamically stable but in moderate respiratory distress, requiring 2-litres/minute of oxygen. Upon transfer to our tertiary/quaternary pediatric hospital, she was placed in an airborne infection isolation room (AIIR) and cared for using airborne droplet and contact precautions. Nebulized hypertonic saline was avoided, and deep nasal suctioning was minimized. She was maintained on low-flow oxygen via nasal prongs for all transfers and placed only on non-invasive ventilation with continuous positive airway pressure (CPAP) through a full-face mask upon arrival in an intensive care AIIR. The chest X-ray (Figure 1) demonstrated increased central interstitial markings with linear and granular opacities. A capillary blood gas demonstrated a respiratory acidosis (pH 7.27; PCO_2_ 50.4; HCO_3_ 22.3). The white blood cell count was normal. An initial nasopharyngeal polymerase chain reaction (PCR) for severe acute respiratory syndrome coronavirus 2 (SARS-CoV-2) was reported as positive (Illness Day 6; cycle threshold [Ct] of 35). On the following day, a multiplex viral PCR was positive for respiratory syncytial virus (RSV), with two subsequent negative SARS-CoV-2 PCRs at 12-hour intervals.

The patient required continuous sedation to tolerate CPAP (minimizing risk of aerosolization and improving CPAP efficacy) and was gradually weaned from respiratory support, then discharged home directly from intensive care after 72 hours (minimizing transmission risk on an inpatient ward). SARS-CoV-2 testing of both parents was negative, but the sibling was not tested. The parents remained with their child, and caution was taken to restrict out-of-room time to a dedicated COVID-19 suspected/confirmed restroom wearing a face mask with strict hand hygiene.

## DISCUSSION

Our report of a critically ill infant with presumed COVID-19 illustrates important pediatric-specific considerations for diagnosis, management, and infection control in the pandemic context. This infant was the first critically ill infant in Canada with a positive SARS-CoV-2 PCR result. This previously healthy infant had no risk factors for critical illness from COVID-19, nor fit the clinical picture of pediatric COVID-19 from epidemiological reports in which nearly none have required intensive care or respiratory support.^1,2^ COVID-19 most frequently produces only mild symptoms in children.^1^ Given the relative rarity of critical illness among pediatric patients with COVID-19, few guidelines exist for this patient population.^3^ Importantly, infants presenting with severe respiratory distress remain far more likely to have common pediatric respiratory syndromes (bronchiolitis, croup, asthma, influenza, bacterial pneumonia) than COVID-19.

This case highlights the diagnostic uncertainty when assessing critically ill children during the COVID-19 pandemic. Despite a presentation consistent with bronchiolitis, the initial positive SARS-CoV-2 PCR shifted the presumptive diagnosis to RSV/SARS-CoV-2 co-infection. Clinicians should avoid premature closure (a form of cognitive bias), searching for other viruses even when faced with a positive SARS-CoV-2 test in the context of severe respiratory disease. In this case, initial nasopharyngeal PCR was positive for SARS-CoV-2 at a Ct value of 35. Higher viral loads are inversely related to Ct values and vary by technique, with values among COVID-19 patients typically between 22–28 and 30–32 for nasopharyngeal and oropharyngeal swabs, respectively.^4^ Multiplex PCR was positive for RSV (Ct of 26), and two subsequent SARS-CoV-2 PCRs were negative. It is possible that this child presented with RSV bronchiolitis, and a low SARS-CoV-2 viral load. Alternatively, the detection of SARS-CoV-2 may have been low-level viral shedding as seen with other ubiquitous respiratory viruses (e.g., rhinovirus), rather than any real clinical contribution by COVID-19. Also, despite high sensitivity and specificity of PCR-based assays in general, a false-positive test result could be possible, further introducing clinical uncertainty.

Adult studies suggest that chest computed tomography is more sensitive than PCR for a diagnosis of COVID-19, and chest imaging abnormalities have been described on computed tomography in children.^5^ However, computed tomography should be avoided for this indication in children, as this would lead to significant ionizing radiation exposure for a majority of children with seasonal viral illness.

Emergency management for bronchiolitis was modified due to presumptive COVID-19. The transmission of SARS-CoV-2 occurs via respiratory droplets and contact with contaminated fomites; airborne spread can occur with aerosol-generating procedures (AGPs). Procedures such as nasopharyngeal suctioning, nebulization-delivered therapies, high-flow nasal oxygen, CPAP, and tracheal intubation are considered AGPs. The advantages of these therapies for bronchiolitis must be balanced against the risk of SARS-CoV-2 aerosolization, particularly when stores of personal protective equipment (PPE) are limited. For therapies deemed necessary, clinicians should don appropriate PPE and proceed. However, nebulized hypertonic saline, for example, may modestly benefit infants with bronchiolitis, but its routine use would be difficult to justify in the pandemic context. The risk of viral dispersion from CPAP depends ultimately on several factors, including flow, duration, and patient cooperation; therefore, the risk of airborne transmission must be weighed against the benefit of avoiding intubation. Ultimately, AGPs should be used very judiciously and, when undertaken, should be performed in AIIR under airborne, droplet, and contact precautions. Emergency management of critically ill children may require departures from pediatric standards of care, such as the separation of child-and-parent to limit nosocomial transmission or limiting blood gas measurements due to additional risks of lab contamination, exposure for personnel, and PPE consumption.

During the COVID-19 pandemic, physicians caring for critically ill children will have to remain vigilant with infection control precautions, while addressing more likely underlying etiologies.

## Consent and reporting

Informed consent was obtained from the family. Manuscript was prepared in accordance with CARE guidelines for case reports (Gagnier JJ, Kienle G, Altman DG, et al.; and CARE Group. The CARE guidelines: consensus-based clinical case report guideline development. *J Clin Epidemiol* 2014;67(1):46-51).
